# Validation and Cross-Cultural Adaptation of the Hindi Version of the Oxford Knee Score in Patients With Knee Osteoarthritis

**DOI:** 10.7759/cureus.23997

**Published:** 2022-04-09

**Authors:** Neeraj K Malhotra, Kavin Khatri, Amit Lakhani, Anshul Dahuja, Deepak Bansal, Ajay Kamat

**Affiliations:** 1 Orthopaedics, Government Medical College, Amritsar, IND; 2 Orthopaedics, All India Institute of Medical Sciences, Bathinda, Bathinda, IND; 3 Orthopaedics, Dr. B. R. (Bhimrao Ramji) Ambedkar State Institute of Medical Sciences, Mohali, IND; 4 Orthopaedics, Guru Gobind Singh Medical College & Hospital, Faridkot, IND; 5 Orthopaedics and Traumatology, AIMC Bassi Hospital, Ludhiana, IND; 6 Orthopaedics and Trauma, Government Medical College, Amritsar, IND

**Keywords:** cross cultural adaptation, osteoarthritis, knee, oks, reliability, hindi, validation

## Abstract

Introduction: Cases of knee osteoarthritis are on the rise in India with an increasingly ageing population. A large number among them shall undergo total knee replacement, so there is a requirement for validated patient-reported outcome measures in the Hindi language. Oxford Knee Score (OKS) is one of the most commonly used patient-reported outcome measure scoring systems. The current study was designed to test and validate cross-cultural adaptation and translate the Hindi version of the Oxford Knee Score (OKS-H).

Material and Methods: The OKS-H was formulated as per recommendations for cross-cultural adaptation and translation. The OKS was tested on 162 patients with knee osteoarthritis who underwent a total knee replacement. Reliability of the OKS-H was tested using the intraclass correlation coefficient (ICC) and internal consistency was assessed using Cronbach’s alpha. The construct validity was assessed using OKS-H, Western Ontario and McMaster Universities Osteoarthritis Index (WOMAC), and 36-Item Short Form Survey (SF-36) questionnaire.

Results: The translation was performed with no major difficulty. The OKS was completed by 158 (97.5%) and 157 (96.9%) patients at test and retest, respectively, after one week. With an ICC of 0.87, OKS had shown good reliability. The construct validity obtained against the WOMAC and SF-36 scores was strong (ICC between 0.49 to 0.86).

Conclusion: The translated OKS-H is a reliable and valid instrument for patient-reported outcome measures in cases of knee osteoarthritis opting for total knee arthroplasty.

## Introduction

Osteoarthritis of the knee is usually associated with pain and the inability to carry out routine activities. It markedly affects mobility and quality of life [1]. Total knee arthroplasty (TKA) is a method of management in end-stage osteoarthritis. The prevalence of osteoarthritis in India varies from 17% to 36% depending on the geographical location within India [2]. It is expected that a large number of patients among these would undergo or consider TKA in the future. It requires tools to measure the success of the outcome and, currently, various instruments are used to evaluate the outcome. Patient-reported outcome measures are being increasingly used in clinical practice. The Oxford Knee Score (OKS) is one such disease-specific, i.e. osteoarthritis, patient-reported outcome measure assessment tool to evaluate the difference between pain along with functionality before and after TKA. It is considered one of the most reliable and valid outcome assessment tools [3]. It is also reported to be an independent predictor of the range of motion in patients after TKA [4]. The OKS is short and comprehensive, and there is no need for a review to obtain objective data. There is minimal observer bias in the measurement of patient satisfaction and expectations after TKA. All these factors have resulted in its extensive use in various studies measuring outcomes in patients after total knee replacement [5-8]. However, language and cultural variations pose a barrier to the successful implementation of this tool for outcome assessment. The Hindi version of the OKS (OKS-H) is available but has not been validated in a prospective manner similar to the original work by Dawson et al. [9]. Moreover, the OKS-H has not been culturally adapted. The primary objective of the study was to translate and adapt the OKS culturally to the Hindi language. In addition, the aim was to test its reliability and validity in patients suffering from knee osteoarthritis undergoing TKA. 

## Materials and methods

Design and sampling

The current study was prospective and observational. Approval for the study was obtained from the ethics committee of AIMC Bassi Hospital, Ludhiana, Punjab, India for clinical research, conducted as per established guidelines under the license from the Clinical Outcomes team at Oxford University Innovation. The OKS-H was developed as described by Guillemin et al. [7]. The original OKS was translated into Hindi by two experienced translators (physiotherapists who had expertise in the management of cases of knee osteoarthritis). Translation work was discussed in a panel with two observers to attain the first preliminary version of OKS-H. The OKS-H was translated back to English by another group of translators (two professional language translators), who were blinded to the study. The agreed version of OKS was completed by 20 patients who were to undergo TKA. They were queried on whether the questions were understandable and to ensure that all the components were without any repetition. Subsequently, a final meeting was held to accomplish the final version (Table 1).

**Table 1:** Oxford Knee Score (English Version)

Today’s Date:										
D	D		M	M		Y	Y	Y	Y	

1	During the past 4 weeks…				
How would you describe the pain you usually have from your knee?					
None		Very mild	Mild	Moderate	Severe
⬜		⬜	⬜	⬜	⬜

2	During the past 4 weeks…				
Have you had any trouble with washing and drying yourself (all over) because of your knee?					
No trouble at all		Very little trouble	Moderate trouble	Extreme difficulty	Impossible to do
⬜		⬜	⬜	⬜	⬜

3	During the past 4 weeks…				
Have you had any trouble getting in and out of a car or using public transport because of your knee? (whichever you tend to use)					
No trouble at all		Very little trouble	Moderate trouble	Extreme difficulty	Impossible to do
⬜		⬜	⬜	⬜	⬜

4	During the past 4 weeks…				
For how long have you been able to walk before pain from your knee becomes severe? (with or without a stick)					
No pain/More than 30 minutes		16 to 30 minutes	5 to 15 minutes	Around the house only	Not at all/pain severe when walking
⬜		⬜	⬜	⬜	⬜

5	During the past 4 weeks…				
After a meal (sat at a table), how painful has it been for you to stand up from a chair because of your knee?					
Not at all painful		Slightly painful	Moderately painful	Very painful	Unbearable
⬜		⬜	⬜	⬜	⬜

6	During the past 4 weeks…				
Have you been limping when walking, because of your knee?					
Rarely/ never		Sometimes, or just at first	Often, not just at first	Most of the time	All of the time
⬜		⬜	⬜	⬜	⬜

7	During the past 4 weeks…				
Could you kneel down and get up again afterwards?					
Yes, easily		With little difficulty	With moderate difficulty	With extreme difficulty	No, impossible
⬜		⬜	⬜	⬜	⬜

8	During the past 4 weeks…				
Have you been troubled by pain from your knee in bed at night?					
No nights		Only 1 or 2 nights	Some nights	Most nights	Every night
⬜		⬜	⬜	⬜	⬜

9	During the past 4 weeks…				
How much has pain from your knee interfered with your usual work (including housework)?					
Not at all		A little bit	Moderately	Greatly	Totally
⬜		⬜	⬜	⬜	⬜

10	During the past 4 weeks…				
Have you felt that your knee might suddenly 'give way' or let you down?					
Rarely/ never		Sometimes, or just at first	Often, not just at first	Most of the time	All of the time
⬜		⬜	⬜	⬜	⬜

11	During the past 4 weeks…				
Could you do the household shopping on your own?					
Yes, easily		With little difficulty	With moderate difficulty	With extreme difficulty	No, impossible
⬜		⬜	⬜	⬜	⬜

12	During the past 4 weeks…				
Could you walk down one flight of stairs?					
Yes, easily		With little difficulty	With moderate difficulty	With extreme difficulty	No, impossible
⬜		⬜	⬜	⬜	⬜

For the validation study, subjects were recruited between May 2016 and February 2020. These were patients who attended our outpatient clinic/outreach physiotherapy center for physical therapy for a six-month period subsequent to surgical procedures. The clinician diagnosed cases of osteoarthritis knee as per the criteria laid down by American Rheumatism Association [8]. The participants' consent was taken, and they were checked for the fulfillment of inclusion criteria. Patients included in the study were cases of knee osteoarthritis aged more than 60 years who underwent TKA. Subjects excluded from the study were those suffering from neurological disease, who could not properly understand or read the Hindi language, cases of cognitive impairment, or pre-existing bony deformity. The patients' demographic characteristics were recorded, including the history of previous joint replacement surgery and comorbid conditions. The sample size was calculated with confirmatory factor analysis. It was estimated that a minimum of 150 patients should be enrolled when a survey using a single feature comprising 12 items is used. The sample size thus calculated allowed for estimating intraclass correlation coefficient (ICC) >0.8 with a precision value of less than 10%.

Measurement scales

The data was collected using the OKS, the Medical Outcome 36-Item Short Form Survey (SF-36) [10], and the Western Ontario and McMaster Universities Osteoarthritis Index (WOMAC) [11]. The OKS includes 12 items: usual level of knee pain, pain on standing up from sitting, trouble to the person with washing and drying clothes, walking time before severe pain, trouble with transport, limping when walking, sensation of knee instability, going out for household/grocery shopping alone, difficulty with kneeling, interference in work subsequent to pain, night pain, and trouble with the use of staircase while going down. The original scoring system had points between 12 (no problem) to 60 (extreme problem) and was used in the current study. 

The SF-36 includes 36 items in eight subscales: physical functioning, role-emotional and mental health, role-physical, bodily pain, general health, vitality, and social functioning. A score of zero denotes the worst health status, while 100 denotes excellent health status. The WOMAC Index is a self-administered questionnaire that includes 24 items in three domains: pain (five items), stiffness (two items), and physical function (17 items). The minimum or best score is zero and the maximum or worst score is 96 points. The total score was obtained after adding scores from each domain. WOMAC is a patient-reported outcome score that has been extensively tested and used in a variety of conditions affecting knee or hip joints [11-12]. The Hindi version of the WOMAC has been validated for use in the Hindi-speaking population [13].

Reliability and internal consistency of OKS-H

In order to check whether items in OKS-H are reproducible or not, ICC was recorded after calculations. It varies from 0 (denoting no agreement) to 1 (signifying absolute agreement). It characterizes the degree to which similar test results are obtained for repetitive calculations when in reality there is no change in the subject during the assessment period. The OKS-H was administered two times with a gap of one week. ICC was measured between the responses to the first and second questionnaires to measure the reliability of the OKS-H. The score was not noted in cases where there were more than three missing responses in the questionnaire. However, if less than three responses were missing, then it was substituted with the mean of completed responses. Internal consistency of OKS was calculated with Cronbach’s alpha [14]. The value of Cronbach’s alpha varies from 0 (no correlation) to 1 (perfect agreement). Values between 0.7 and 0.9 denote strong internal consistency with moderate correlation; higher values may not actually be useful and indicate redundancy of items [15]. Test-retest reliability to look out for stability of instrument with time was checked by involving 40 outpatients. The interval between testing and retesting reliability was one week to ensure a relatively stable clinical condition. 

Construct validity testing

Construct validity is the ability of a scale to measure accurately what it is supposed to measure. In the present study, Spearman’s correlation coefficient was used to ascertain correlation strength between translated OKS-H, WOMAC score, and SF-36. We had hypothesized about achieving a strong correlation between the scales. 

Responsiveness testing

It is the ability of the questionnaire to detect clinically important changes with time, even though the changes are small. Standardized response mean and effect size was calculated using the responsiveness of the OKS-H. The patients were followed up for one year as TKA patients are expected to achieve maximum recovery by then [16].

Floor and ceiling effect

In order to determine the floor and ceiling effect of the OKS-H, the proportion of entries with the highest and lowest possible scores were noted. Dawson et al. [9] had described the lowest score as the best and the highest being the worst, so we had defined low score as ceiling and high score as floor. Improvement or deterioration may not be detected in patients with the highest or lowest score in the spectrum. The effect was noted in cases where more than 15% of the subjects attained extreme scores [17].

Cross-cultural adaptation

The second version or the version translated back by the translators of the OKS-H was used in the validation study as it was understandable by the subjects and accepted in the pretest. 

Statistical analysis

The categorical data were described as frequency and percentage values while quantitative variables were described using mean and standard deviation. The confidence interval was set at 95%. SPSS for Windows, Version 16.0 (Released 2007; SPSS Inc., Chicago, United States) was used for statistical analysis, and a p-value of 0.05 was considered to be significant.

Cronbach's alpha was used to estimate internal consistency, and the ICC was used for test-retest reliability. The construct validity of the scale is indicated by divergent and convergent validity. The nonparametric data were assessed using Spearman’s rank correlations. The correlation is graded as strong (>0.5), moderate (0.35 to 0.5), and weak (<0.35). Divergent validity is expected in lower correlation and vice versa true from convergent validity. Exploratory factor analysis was used to assess dimensionality.

## Results

Cross-cultural adaptation

In the pretest, the second preliminary OKS-H was accepted. The items in the questionnaire were understood to a satisfactory extent by the participants.

Validation

Participants

The characteristics of the participants are presented in Table 2. A total of 162 patients were enrolled in the study and subjected to internal consistency and validity. Among them, 65 (40.12%) were included for the assessment of reliability. There was no recording of data missed for an item of the OKS and the SF-36 questionnaire [9].

**Table 2:** Patient demographics *Categorical variables are represented as number (percentage) #Quantitative variables are represented as mean±standard deviation

Characteristics	Total participants (n=162)^#^		Reproducibility group (n=65)^#^		
Age (in years)	60.4±5.4		62.5±6.1		
Gender	Male	Female	Male		Female
	68	94	14		51
Duration of osteoarthritis (years)	10±1.4		9±1.7		
Body mass index (kg/m^2^)	24±3.4		25±4.4		
Comorbid conditions					
Diabetes	24 (15.4)		9 (13.5)		
Hypertension	56 (35.1)		25 (39)		
Cardiovascular	31 (19.6)		11 (18)		
Other	20 (12.3)		6 (9.1)		
Living					
Rural	104 (64.7)		45 (70.2)		
Urban	58 (36.9)		20 (30.7)		
House					
With stairs	31 (19.3)		15 (23.6)		
Without stairs	131 (81.4)		50 (77.6)		
Aid while walking	142 (88.7)		55 (85.6)		
OKS score	41±4.1		43.6±3.4		
SF-36 subscale score					
Bodily pain	24.6±18.9		26.4±16.8		
Role -physical	20.4±21.8		24.3±22.3		
Physical functioning	23.5±18.5		27.4±15.8		
General health	44.7±23.8		42.4±26.7		
Social functioning	45.4±24.9		42.4±21.8		
Mental health	48.4±26.7		50.4±23.7		

Internal Consistency

The internal consistency measured with Cronbach’s alpha was good at 0.883. 

Reliability

The OKS-H was completed by 158 (97.5%) and 157 (96.9%) patients at test and retest, respectively. The mean scores recorded after the test and retest of OKS-H were 29.4±7.2 and 30.1±7.6, respectively. The difference between the two scores of each item in OKS-H was not statistically significant (Table 3). With an ICC of 0.91 (95% CI, 0.87-0.97), there was high reliability with OKS-H.

**Table 3:** Reliability of Hindi version of Oxford Knee Score

Item No.	Item	Test			Retest			P value
		Mean with SD	Item total correlation	Alpha if item is removed	Mean with SD	Item total correlation	Alpha if item is removed	
1	Usual level of pain	2.1±0.93	0.67	0.86	1.9±0.89	0.71	0.89	<0.001
2	Trouble with washing and drying	1.65±0.89	0.57	0.9	1.68±01.20	0.62	0.92	<0.001
3	Trouble with transport	1.68±0.91	0.65	0.89	1.71±0.98	0.73	0.85	<0.001
4	Walking duration before severe pain	1.87±0.88	0.71	0.91	2.15±0.94	0.67	0.89	<0.001
5	Pain on standing up from sitting	2.12±0.98	0.73	0.87	1.98±0.88	0.81	0.92	<0.001
6	Limping when walking	1.78±0.94	0.76	0.95	1.67±0.87	0.69	0.88	<0.001
7	Difficulty in kneeling	0.94±1.11	0.59	0.89	0.98±1.2	0.76	0.93	<0.001
8	Pain in bed at night	1.54±0.76	0.69	0.92	1.54±0.76	0.69	0.92	<0.001
9	Work interference due to pain	2.15±1.23	0.72	0.94	2.19±1.13	0.74	0.91	<0.001
10	Sense of knee instability	1.50±0.98	0.67	0.88	1.47±0.95	0.73	0.93	<0.001
11	Doing household/shopping alone	1.45±0.95	0.8	0.91	1.47±0.97	0.81	0.95	<0.001
12	Trouble with walking down stairs	2.34±1.13	0.68	0.88	228±1.04	0.74	0.9	<0.001

Construct validity

The prior assumption of convergent and divergent validity, correlations between SF-36, OKS-H, and WOMAC were confirmed to be significant with moderate to strong correlations (r > 0.35) (Table 4). 

**Table 4:** Construct Validity of OKS-H Scale OKS-H: Oxford knee score, Hindi version; SF-36: 36-Item Short Form Survey (SF-36); WOMAC: Western Ontario and McMaster Universities Osteoarthritis Index

Scale	Mean ± SD	OKS-H r (correlation coefficient)	p-value
SF-36 subscales			
Physical	30±15	0.55 (0.39-0.63)	<0.001
Bodily Pain	39±17	0.47 (0.36-0.63)	<0.001
General Health	35±16	0.50 (0.31-0.59)	<0.001
Social functioning	48±17	0.58 (0.42-0.68)	<0.001
Role-Emotional	52±15	0.41 (0.25-0.55)	<0.001
Mental Health	47±23	0.47 (0.27-0.64)	<0.001
Role-Physical	41±19	0.56 (0.43-0.69)	<0.001
Vitality	43±17	0.35 (0.14-0.53)	<0.001
WOMAC			
Stiffness	5±3	-0.53 (-0.73 to -0.42)	<0.001
WOMAC Pain Function	9±4	-0.70 (-0.81 to -0.53)	<0.001
Function	34±13	-0.74 (-0.84 to -0.58)	<0.001

Floor and ceiling effects

The maximum or worst score was recorded by only a small proportion of the patients, so a low floor effect for the OKS-H questionnaire was recorded. The low or minimal scores were reported by more than 17% of the patient for 11 of the 12 items on the scale. 

## Discussion

Culturally, the Indian population is different from the western population in terms of distinct lifestyle and linguistic features. In our study, 68% of participants lived on a property without an elevator, and 45% used squat toilets. This specific subset of the population is increasing at a fast pace and ageing rapidly, hence the demand for TKA is expected to rise markedly. There are a number of tools for the assessment of patient-reported outcomes measures after TKA, but OKS, due to its ease of use, wide reporting, and validation in various languages has resulted in its emergence as a favourite tool. 

The procedure to translate and culturally adapt the OKS was successful, and a fairly comprehensible OKS-H was obtained. In literature also, minimal problems have been stated in using translated versions of the OKS [18-20]. The OKS-H was confirmed to be internally consistent as Cronbach’s alpha coefficient was high and acceptable. Similar internal consistency was reported by previous studies [21-22]. In terms of reliability of OKS-H, the results were almost similar to those obtained by the other language versions of OKS in spite of using varied time intervals for the administration of scale. The Dutch version reported an ICC of 0.97 (for interval of one week), the Italian 0.88 (for interval of three to five days), and the Swedish 0.94 (for interval of four weeks). 

In the current study, cross-cultural adaptation and translation of the OKS to the Hindi language were carried out, and psychometric properties like validity, internal consistency, and reliability in cases of TKA were examined. The results demonstrated good psychometric properties with the OKS-H. WOMAC and SF-36 subscales correlated with the OKS-H. The results were similar in other language versions of OKS reported in the literature [18-20].

The current study has some limitations. First, our patients were recruited from regional hospitals in northern India, so the generalization of the findings to the larger national population may be limited. Secondly, limited sample size restricts subgroup analysis. So, a larger cohort would be required to study this issue. 

## Conclusions

The OKS-H has suitable psychometric properties with respect to reliability and validity in patients suffering from osteoarthritis who underwent total knee replacement and is in agreement with the widely used original OKS version. It is well accepted by patients, and its incorporation into clinical practice would result in a better assessment of patients' perception of health-related quality of life and the outcome of health interventions directed at them. Furthermore, the current study would provide a valuable basis for conducting more studies concerning patient self-assessed scores in Asian languages.
